# Occurrence of fatal infective endocarditis: a population-based study in Finland

**DOI:** 10.1186/s12879-019-4620-0

**Published:** 2019-11-21

**Authors:** Elina Ahtela, Jarmo Oksi, Jussi Sipilä, Päivi Rautava, Ville Kytö

**Affiliations:** 1 Heart Center, Turku University Hospital and University of Turku, PO Box 52, 20521 Turku, Finland; 2 Department of Infectious Diseases, Turku University Hospital and University of Turku, Turku, Finland; 3 Siun sote, North Karelia Central Hospital, Joensuu, Finland; 4 Division of Clinical Neurosciences, Turku University Hospital, Turku, Finland; 5 Department of Neurology, University of Turku, Turku, Finland; 6 Department of Public Health, University of Turku, Turku, Finland; 7 Turku Clinical Research Centre, Turku University Hospital, Turku, Finland; 8 Research Center of Applied and Preventive Cardiovascular Medicine, University of Turku, Turku, Finland; 9 Center for Population Health Research, Turku University Hospital and University of Turku, Turku, Finland; 10 Administrative Center, Hospital District of Southwest Finland, Turku, Finland

**Keywords:** Cause of death, Epidemiology, Incidence rate, Infective endocarditis

## Abstract

**Background:**

Infective endocarditis (IE) is a serious mainly bacterial infection associated with high mortality. Epidemiology of fatal IE is however largely unknown. We studied occurrence and trends of fatal IE in a population-based setting.

**Methods:**

All adults (≥18 years of age) who deceased due to IE in Finland during 2004–2016 were studied. Data was collected from the nationwide, obligatory Cause of Death Registry. Background population consisted of 28,657,870 person-years and 651,556 deaths.

**Results:**

Infective endocarditis contributed to death in 754 cases and was the underlying cause of death in 352 cases. The standardized incidence rate of deaths associated with IE was 1.42 (95% confidence interval (CI): 1.32–1.52) per 100,000 person-years. Incidence rate increased progressively with aging from 50 years of age. Men had a two-fold risk of acquiring fatal infective endocarditis compared to women (risk ratio (RR) 1.95; 95% CI: 1.71–2.22; *P* < 0.0001). On average, IE contributed to 1.16 (95% CI: 1.08–1.24) out of 1000 deaths in general adult population. The proportionate amount of deaths with IE was highest in population aged < 40 years followed by gradual decrease with aging. Incidence rate and proportion of deaths caused by IE remained stable during the study period.

**Conclusions:**

Our study describes for the first time the population-based epidemiology of fatal IE in adults. Men had a two-fold risk of acquiring fatal IE compared to women. Although occurrence of fatal IE increased with aging, the proportion of deaths to which IE contributed was highest in young adult population.

## Background

Infective endocarditis (IE) is a serious mainly bacterial infection associated with high mortality. Incidence of IE admissions has previously found to be 2–8/100,000 person-years [1–9]. Currently knowledge on occurrence of fatal IE is based on follow-up studies of hospitalized patients with short-term mortality (i.e. within 30 days or during the hospital stay) reported to be 10–24% [3, 5, 7, 10] and longer-term (i.e. within 6 months–1 year from the diagnosis) mortality 22–37% [7, 8, 11, 12] after IE diagnosis. Overall mortality rate after IE admission has remained stable over the years [5, 10, 13]. Knowledge on occurrence of non-hospitalized fatal IE cases is however scarce and population-based epidemiology of fatal IE is thus unknown. The purpose of this nationwide population-based study was to investigate sex- and age-specific differences and temporal trends in the occurrence of fatal IE.

## Methods

Adults (aged ≥18 years) with IE-related death during 2004–2016 were studied. Deceased with IE contributing to death recorded in death certificate were retrospectively identified from the nationwide Cause of Death Registry of Statistics Finland (Helsinki, Finland). Issuance of death certificate with determination of the causes of death and collection of death certificates into Statistics Finland database is required by law, and has a complete coverage of population. The physician issuing the death certificate determines the one cause of death he/she deems is the underlying cause of death, and this is recorded as the official cause of death based on World Health Organization (WHO) classification. In addition, other causes contributing to death are recorded. All death certificates are verified by the competent authority in charge of forensic medicine.

Infective endocarditis was recognized by International Classification of Diseases, Tenth Revision (ICD-10) codes I33, I38 or I39. Age- and sex-specific population and mortality data of the Finnish population from the years 2004–2016 were obtained from Statistics Finland [14]. The study period included 28,657,870 person-years and 651,556 total deaths. The person-years of each study year were estimated by population at the end of the year. The study was approved by the National Institute for Health and Welfare (permission no. THL/1484/5.05.00/2017) and Statistics Finland (TK53–1410-15).

Age- and sex-specific incidence rates and proportions of death were calculated. Standardization of incidence was performed with direct method and European 2013 standard population. Differences in continuous variables were analyzed by using a t-test. The associations of age, sex and study year with the incidence rate and proportion of deaths were studied by using Poisson regression modeling. Incidence rate and proportionate amount of deaths were modelled with using the logarithm of corresponding population or number of deaths as an offset parameter [14]. Impact of age on associations with sex were studied with interaction analysis. Monthly and seasonal (winter: December–February; spring: March–May; summer June–August; autumn: September–November) variations in the number of deaths due to IE were analyzed with Chi-Squared test. Ninety-five percent confidence intervals (95% CI) of count variables were calculated assuming Poisson distribution. Statistical significance was inferred at *P* < 0.05. All *P*-values were two-sided. The SAS system version 9.4 (SAS Institute Inc.) was used for statistical analyses.

## Results

Infective endocarditis contributed to death in 754 cases during 2004–2016. The mean age of deceased with IE was 68.6 (standard deviation (SD) 16.7 years) (median 68.6; range: 20–99 years). Women (*n* = 306) were older (mean age 73.1 years (SD 15.8)) than men (*n* = 448; mean age 65.6 years (SD 16.6); *P* < 0.0001). Infective endocarditis-associated death occurred in hospital or other healthcare facility in 89.5%, at home in 8.6% and elsewhere (including institutional care and housing services in social care and retirement homes) in 1.6% of cases, while 0.3% of deaths occurred abroad. There were no monthly (*P* = 0.642) or seasonal (*P* = 0.214) differences in occurrence of IE-associated deaths.

**Fig. 1:**
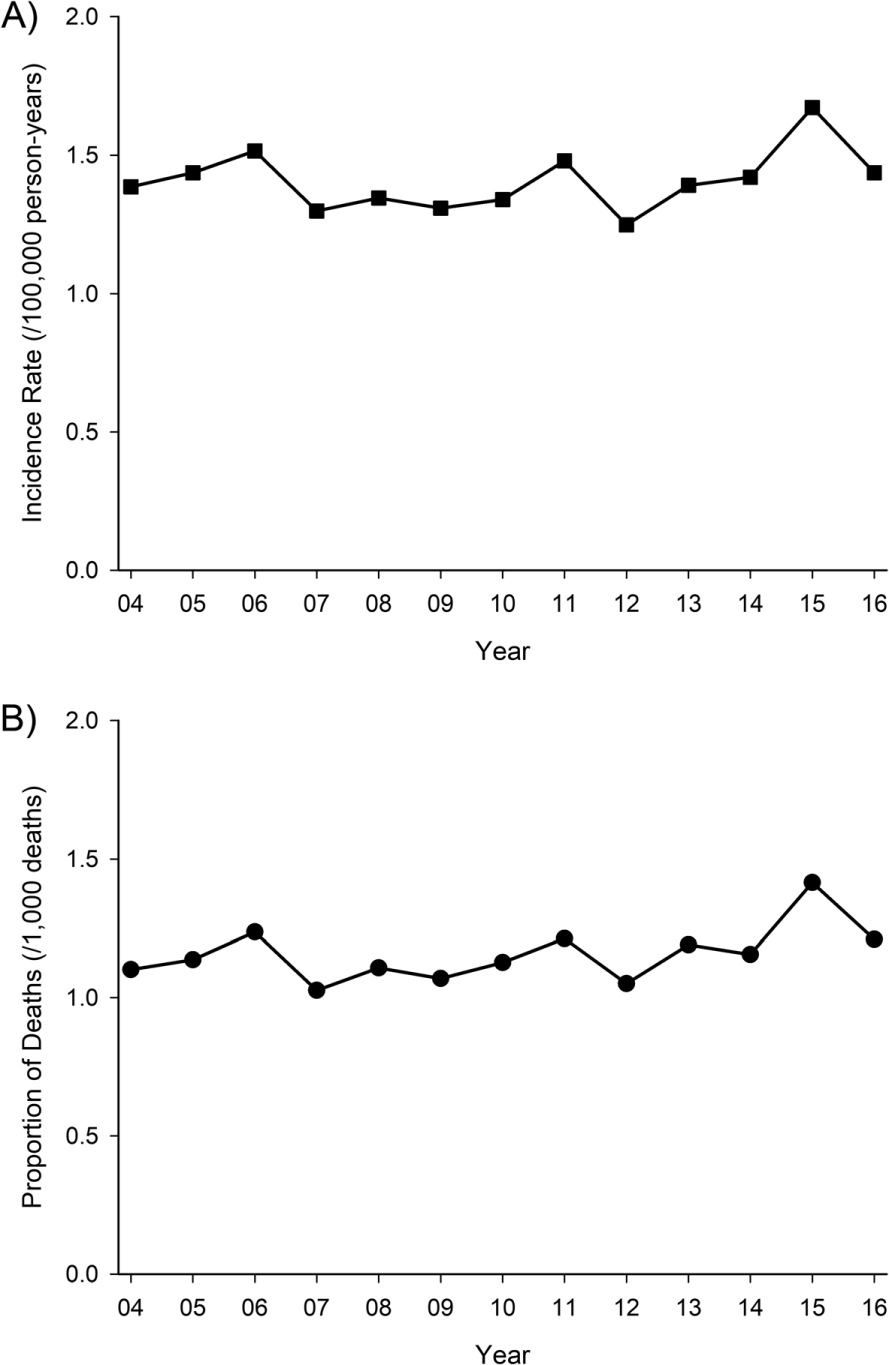
Occurrence of deaths associated with infective endocarditis (IE) in adult population in Finland during 2004–2016. Standardized incidence rate (**a**) and proportion of deaths (**b**) associated with IE from total deaths. No significant annual trend was observable for incidence rate (**a**) or proportionate amount of deaths (**b**) due to IE

The standardized incidence rate of IE-associated deaths in total adult population was 1.42 (95% CI: 1.32–1.52) per 100,000 person-years. Occurrence was significantly age-dependent (*P* < 0.0001). Incidence rate was lowest in population aged 18–29 years, remained similar in population aged 30–49, and increased progressively with aging from 50 years of age (Table 1). In the oldest population (≥90 years), the likelihood of acquiring IE severe enough to contribute to death was 37-fold (8.83 deaths/100,000) compared to the youngest population. Men had a two-fold risk of acquiring fatal infective endocarditis compared to women (risk ratio (RR) 1.95; 95% CI: 1.71–2.22; *P* < 0.0001). Association was not modified by age (interaction *P* = 0.102). Overall incidence rate was 1.95 (95% CI: 1.79–2.13)/100,000 person-years in men and 0.99 (95% CI: 0.88–1.12)/100,000 in women. Incidence rate of IE-associated deaths had no significant trend of change (*P* = 0.994) during the study period (Fig. 1).

**Table 1:** Incidence Rate of Deaths Associated With Infective Endocarditis in Adult Population of Finland during 2004–2016

	Men		Women		Total	
Age (Years)	N	Incidence rate (95%CI)^a^	N	Incidence rate (95%CI)^a^	N	Incidence rate (95%CI)^a^
18–29	17	0.32 (0.19–0.51)	8	0.16 (0.07–0.31)	25	0.24 (0.16–0.35)
30–39	28	0.63 (0.42–0.91)	12	0.28 (0.15–0.49)	40	0.46 (0.33–0.62)
40–49	29	0.61 (0.41–0.88)	8	0.18 (0.08–0.34)	37	0.40 (0.28–0.55)
50–59	61	1.22 (0.94–1.57)	26	0.52 (0.34–0.76)	87	0.87 (0.70–1.07)
60–69	102	2.45 (2.00–2.98)	38	0.85 (0.60–1.17)	140	1.63 (1.37–1.92)
70–79	118	5.01 (4.15–6.00)	88	2.89 (2.32–3.56)	206	3.81 (3.31–4.37)
80–89	81	8.62 (6.85–10.72)	97	5.22 (4.23–6.37)	178	6.36 (5.46–7.37)
90-	12	11.80 (6.10–20.61)	29	8.00 (5.36–11.49)	41	8.83 (6.34–11.98)
Total Crude	448	1.65 (1.50–1.81)	306	1.07 (0.95–1.19)	754	1.35 (1.26–1.45)
Standardized		1.95 (1.79–2.13)		0.99 (0.88–1.12)		1.42 (1.32–1.52)

*Abbreviation*: *CI* confidence interval

^a^Per 100,000 person-years

On average, IE contributed to 1.16 (95% CI: 1.08–1.24) out of 1000 deaths in general adult population. The proportionate amount of deaths with IE was highest (4.42 (95% CI: 3.41–5.64)/1000) in adult population aged < 40 years followed by gradual decrease with aging down to 0.39 (95% CI: 0.28–0.53)/1000 in nonagenarians (*P* < 0.0001; Table 2). Men had higher rate of deaths associated with IE (1.38 (95% CI: 1.25–1.51)/1000) compared to women (0.94 (95% CI: 0.84–1.05)/1000) with RR of 1.47 (95% CI: 1.17–1.84; *P* = 0.0008). Association was not modified by age (interaction *P* = 0.234). Proportion of deaths associated with IE remained stable (*P* = 0.465) during the study period (Fig. 1).

**Table 2:** Proportion of Deaths Associated With Infective Endocarditis in Adult Population of Finland during 2004–2016

	Men		Women		Total	
Age (Years)	N	Proportion (95%CI)^a^	N	Proportion (95%CI)^a^	N	Proportion (95%CI)^a^
18–29	17	3.44 (2.00–5.51)	8	4.85 (2.10–9.57)	25	3.79 (2.46–5.60)
30–39	28	4.70 (3.12–6.79)	12	5.58 (2.88–9.75)	40	4.93 (3.52–6.72)
40–49	29	2.08 (1.40–2.99)	8	1.34 (0.58–2.64)	37	1.86 (1.31–2.56)
50–59	61	1.71 (1.31–2.20)	26	1.60 (1.05–2.35)	87	1.68 (1.34–2.07)
60–69	102	1.64 (1.33–1.99)	38	1.25 (0.88–1.71)	140	1.51 (1.27–1.78)
70–79	118	1.39 (1.15–1.67)	88	1.50 (1.20–1.85)	206	1.44 (1.25–1.65)
80–89	81	0.88 (0.70–1.10)	97	0.73 (0.60–0.90)	178	0.80 (0.68–0.92)
90-	12	0.47 (0.24–0.81)	29	0.37 (0.25–0.53)	41	0.39 (0.28–0.53)
Total	448	1.38 (1.25–1.51)	306	0.94 (0.84–1.05)	754	1.16 (1.08–1.24)

*Abbreviation*: *CI* confidence interval

^a^Per 1000 deaths

Infective endocarditis was the underlying cause of death in 352 cases (46.7%). Septicemia was recorded to be the underlying cause of death in 11.5% of cases and other infectious disease in 2.8% (Table 3). Of septicemias as the underlying cause of death, 62.1% were recorded to be caused by staphylococci, 19.5% by streptococci and 18.4% by other or undetermined pathogens. Of the staphylococcal septicemias, 91.3% were caused by *Staphylococcus aureus*. Cardiovascular diseases were the leading non-infectious underlying causes of death (16.3%) with coronary artery disease/myocardial infarction (39.8%) and cerebrovascular disease/stroke (18.7%) as major contributors. Valvular stenosis or insufficiency was the underlying cause of death in 46 cases (6.1%) with aortic valve affected in 60.9% (of which 89.2% were stenosis), mitral valve in 23.9% (stenosis in 63.6%) and tricuspid valve in 6.5%.

**Table 3:** Underlying Causes of Deaths Associated With Infective Endocarditis in Finland during 2004–2016

Underlying cause of death	N	%
Endocarditis	352	46.7
Septicemia	87	11.5
Other infection	21	2.8
Cardiovascular/circulatory disease^a^	123	16.3
Neoplasm/blood disease^a^	48	6.4
Psychiatric disease^a^	30	4.0
Digestive tract disease^a^	25	3.3
Endocrinological disease^a^	18	2.4
Musculoskeletal/Connective tissue disease^a^	17	2.7
External causes/Accidents	12	1.6
Congenital malformations	6	0.8
Respiratory tract disease^a^	6	0.8
Nervous system disease^a^	3	0.4
Skin/Subcutaneous tissue disease^a^	3	0.4
Genitourinary disease^a^	3	0.4

^a^Excluding infections

The standardized incidence rate of deaths with IE as the underlying cause was 0.66 (95% CI: 0.59–0.73)/100,000 in total adult population. Age distribution of deaths with IE as the underlying cause was similar to all deaths associated with IE (Additional file 1: Table S1). Sex difference in deaths with IE as the underlying cause (men vs. women RR 2.21; 95% CI: 1.72–2.83; *P* < 0.0001) was also similar to all deaths associated with IE (Additional file 1: Table S1). Proportion of deaths with IE as the underlying cause was 0.54 (95% CI: 0.49–0.60)/1000 deaths in total adult population with age- and sex distribution comparable to all deaths associated with IE (Additional file 1: Table S2).

## Discussion

Currently knowledge on IE is based on studies of hospitalized patients, but little is known on total IE occurrence. In this population-based study we studied the total occurrence of fatal IE. The standardized incidence rate of deaths associated with IE in total adult population was 1.42 per 100,000 person-years. Incidence rate was lowest in population aged 18–29 years, remained similar in population aged 30–49, and increased progressively with aging from 50 years of age. This is in line with previous findings on increasing rate of admissions with IE [6, 8, 11] and mortality after admission with IE with aging [4, 7, 11, 15]. Increasing risk of fatal IE with aging is likely associated with higher prevalence of predisposing degenerative valve diseases and comorbidities [16]. We found the oldest (≥90 years) population to have 37-fold likelihood of acquiring fatal IE compared to young adults. The proportionate amount of deaths with IE was highest (4.42/1000) in adult population aged < 40 years followed by gradual decrease with aging. This underlines both the severity of IE also in younger adults and the fact that morbidity in general increases with aging.

We found that incidence rate of fatal IE had no significant trend of change during the study period. Furthermore, we found that proportion of deaths associated with IE remained stable during the study period. Previous studies have comparably found that mortality of IE has not decreased over the years [5, 10, 13]. Reason for the non-decreasing IE mortality is unclear. It is possible that improved diagnostic methods have enabled to make diagnoses in more severely ill patients than previously. Also, changes in microbiological etiology and demographics of IE population, i.e. increasing proportion of younger IE patients [17] are likely contributors.

Previous studies have found male overrepresentation (9–20%) among patients with IE [5–8, 18], but population-level difference for risk of fatal IE between sexes has remained unknown. In the current study men had a two-fold risk of acquiring fatal IE compared to women. This difference was not modified by age. Furthermore, men had 1.5-fold likelihood of death to be associated with IE compared to women. One possible explanation for male predominance in IE is the higher prevalence of risk factors [9] and predisposing medical conditions, such as degenerative valve disease, for IE in men. Moreover, the intravenous drug use, a well-known risk factor of IE, is more common in men [19, 20]. These factors might also correlate to the increased mortality of men with IE compared to the women. A study from UK found the risk of dying during IE hospital admission to be significantly higher in men compared to women [9]. Interestingly, studies from Belgium, Spain, the Netherlands and Italy have found mortality after admission due to IE to be higher in women compared to men [12, 15, 18, 21]. A prospective observational cohort study from Spain involving patients with native-valve IE found female sex to be a predictor of death in univariate analysis but not in multivariate analysis [22]. Sex differences in patients with IE thus require further study.

In our study infective endocarditis-associated death occurred in hospital or other healthcare facility in 89.5% and at home in 8.6% of cases. Individuals without IE diagnosis prior to death most likely died outside the hospital or other healthcare facility, e.g. at home. The incidence rate for IE hospital admissions in Finnish adult population is found to be 6.33/100,000 and 30-day mortality after admission to be 11.3% [17]. Previously, the in-hospital mortality of patients with IE has reported to be 14–25% [7, 10, 23]. Current finding of 1.42/100,000 incidence rate of fatal IE in total thus suggests that significant portion of IE-related deaths are likely to occur later than a month after IE admission or to patients without IE diagnosis when alive. Further studies on long-term outcomes after IE admissions are however warranted.

We found that in IE-related deaths septicemia was the underlying cause of death in 11.5% of cases. Patients with IE often have uncontrolled infection and complications of sepsis (e.g. septic shock) might lead to death. In these cases septicemia might be coded as the underlying cause of death and IE as a cause contributing to death.

Infective endocarditis, septicemia or other infection was deemed as underlying cause of death in 61% and valvular heart disease in 6.1% of deaths associated with IE. Valvular heart disease is a known risk factor for IE. Although determination of the underlying cause of death may be prone to subjective interpretation of findings, our results suggest that significant valvular disease was the cause of death related to IE in 6% of the cases.

Occurrences of infectious diseases including sepsis [24], pneumonia [25] and appendicitis [26] are found to have seasonal variation. Seasonal changes in pathogen and host factors may create seasonal surges in disease incidence, which may become increasingly important in the context of global climate change [27]. In addition to severe bacterial infections, cardio- and cerebrovascular diseases are also associated with seasonal variation [28, 29]. Curiously however, we found no seasonal or monthly variation in occurrence of fatal IE in the current study. As noted previously, most deaths due to IE occur later than 1 month after admission due to IE and furthermore probably after the acute phase of the infection. This could explain, in part, the lack of seasonal variation in occurrence of fatal IE.

To our knowledge this is the first population-level study on the occurrence of fatal IE. The current study is based on nationwide, obligatory database with a complete population coverage. There are however limitations to the study. Usage of registry data has certain limitations as we did not have the access to the more detailed personal level data, e.g. microbiological, clinical or autopsy data. The general autopsy rate of all deaths in Finland during the study period was 28.4% compared to mean of 16.6% in all members of the European Union [30]. The diagnoses were made by individual physicians and pathologists which may influence the results. Accuracy of used ICD-10 IE codes is however good among hospitalized patients with our previous study showing Duke criteria [31] specificity of 96.8% [17] while previous investigation of comparable design found 100% specificity and 90% sensitivity [32]. Moreover, we did not have the data of the possible surgical procedures that might influence the mortality or information on time from possible IE diagnosis to death. Additionally, the death certificates might contain coding errors. The gold standard of the diagnostics of IE in Finland are the modified Duke criteria [31]. The ICD-10 codes for IE include both possible and definite IE. Thus, some of the diagnoses of IE, especially in the oldest population group, might be uncertain, as the use of specific diagnostic tools, such as transesophageal echocardiography, are not as frequently used.

## Conclusions

In conclusion, our study describes the population-based epidemiology of fatal IE in adults for the first time. Infective endocarditis-associated death occurred in average to 1.4 out of 100,000 person-years and contributed to 1.2 of 1000 deaths in adult population. Men had a two-fold risk of acquiring fatal IE compared to women. Occurrence of fatal IE increased progressively with aging from 50 years of age, but the proportion of deaths to which IE contributed was highest in young adult population. Incidence rate of fatal IE remained stable over time.

## Supplementary information

**Additional file 1: Table S1.** Incidence Rate of Deaths with Infective Endocarditis as the Underlying Cause in Adult Population of Finland during 2004–2016. **Table S2.** Proportion of Deaths with Infective Endocarditis as the Underlying Cause in Adult Population of Finland during 2004–2016.

## Data Availability

The data and study materials will be made available to those who fulfil the requirements of applicable Finnish laws and regulations for purposes of reproducing the results or replicating the procedure. Contact person: Corresponding author.

## Abbreviations

CI: Confidence interval
ICD-10: International Classification of Diseases, Tenth Revision
NICE: The National Institute for Health and Clinical Excellence
RR: Risk ratio
SD: Standard deviation
