# Supplementary material for: Occurrence of fatal infective endocarditis: a population-based study in Finland
Source: BMC Infect Dis. 2019 Nov 21;19:987. doi: 10.1186/s12879-019-4620-0 (PMC6873758; doi:10.1186/s12879-019-4620-0)
Supplement: Supplementary file 1 — Additional file 1: Table S1. Incidence Rate of Deaths with Infective Endocarditis as the Underlying Cause in Adult Population of Finland during 2004–2016. Table S2. Proportion of Deaths with Infective Endocarditis as the Underlying Cause in Adult Population of Finland during 2004–2016. [file 12879_2019_4620_MOESM1_ESM.docx]

**Table S1.** Incidence Rate of Deaths With Infective Endocarditis as the Underlying Cause in Adult Population of Finland during 2004–2016.

|  | **Men** | | **Women** | | **Total** | |
| --- | --- | --- | --- | --- | --- | --- |
| Age (Years) | N | Incidence rate (95%CI)* | N | Incidence rate (95%CI)* | N | Incidence rate (95%CI)* |
| 18-29 | 8 | 0.15 (0.06-0.30) | 4 | 0.08 (0.02-0.20) | 12 | 0.12 (0.06-0.20) |
| 30-39 | 10 | 0.22 (0.11-0.41) | 2 | 0.05 (0.01-0.17) | 12 | 0.14 (0.07-0.24) |
| 40-49 | 18 | 0.38 (0.23-0.60) | 3 | 0.07 (0.01-0.19) | 21 | 0.22 (0.14-0.34) |
| 50-59 | 35 | 0.70 (0.50-0.98) | 9 | 0.18 (0.08-0.34) | 44 | 0.44 (0.32-0.59) |
| 60-69 | 49 | 1.18 (0.87-1.56) | 17 | 0.38 (0.22-0.61) | 66 | 0.77 (0.59-0.98) |
| 70-79 | 52 | 2.21 (1.65-2.89) | 43 | 1.41 (1.02-1.90) | 95 | 1.75 (1.42-2.15) |
| 80-89 | 41 | 4.37 (3.13-5.92) | 42 | 2.26 (1.63-3.06) | 83 | 2.97 (2.36-3.68) |
| 90- | 6 | 5.90 (2.17-12.84) | 13 | 3.59 (1.91-6.13) | 19 | 4.09 (2.46-6.39) |
| Total Crude | 219 | 0.81 (0.71-0.92) | 133 | 0.46 (0.39-0.55) | 352 | 0.63 (0.57-0.70) |
| Standardized |  | 0.95 (0.84-1.07) |  | 0.43 (0.36-0.52) |  | 0.66 (0.59-0.73) |

**Abbreviation**: CI, confidence interval

* Per 100,000 person-years.

**Table S2.** Proportion of Deaths With Infective Endocarditis as the Underlying Cause in Adult Population of Finland during 2004–2016.

|  | **Men** | | **Women** | | **Total** | |
| --- | --- | --- | --- | --- | --- | --- |
| Age (Years) | N | Proportion (95%CI)* | N | Proportion (95%CI)* | N | Proportion (95%CI)* |
| 18-29 | 8 | 1.62 (0.70-3.19) | 4 | 2.43 (0.66-6.21) | 12 | 1.82 (0.94-3.18) |
| 30-39 | 10 | 1.68 (0.80-3.09) | 2 | 0.93 (0.11-3.36) | 12 | 1.48 (0.76-2.58) |
| 40-49 | 18 | 1.29 (0.77-2.04) | 3 | 0.50 (0.10-1.47) | 21 | 1.06 (0.65-1.61) |
| 50-59 | 35 | 0.98 (0.68-1.37) | 9 | 0.56 (0.25-1.05) | 44 | 0.85 (0.62-1.14) |
| 60-69 | 49 | 0.79 (0.58-1.04) | 17 | 0.56 (0.33-0.89) | 66 | 0.71 (0.55-0.90) |
| 70-79 | 52 | 0.61 (0.46-0.80) | 43 | 0.73 (0.53-0.99) | 95 | 0.66 (0.54-0.81) |
| 80-89 | 41 | 0.45 (0.32-0.61) | 42 | 0.32 (0.23-0.43) | 83 | 0.37 (0.30-0.46) |
| 90- | 6 | 0.23 (0.09-0.51) | 13 | 0.17 (0.09-0.28) | 19 | 0.18 (0.11-0.28) |
| Total | 219 | 0.67 (0.59-0.77) | 133 | 0.41 (0.34-0.48) | 352 | 0.54 (0.49-0.60) |

**Abbreviation**: CI, confidence interval

* Per 1,000 deaths
